# Spectral Fingerprinting of Individual Cells Visualized by Cavity-Reflection-Enhanced Light-Absorption Microscopy

**DOI:** 10.1371/journal.pone.0125733

**Published:** 2015-05-07

**Authors:** Yoshiyuki Arai, Takayuki Yamamoto, Takeo Minamikawa, Tetsuro Takamatsu, Takeharu Nagai

**Affiliations:** 1 The Institute of Scientific and Industrial Research, Osaka University, Osaka, Japan; 2 Department of Pathology and Cell Regulation, Graduate School of Medical Science, Kyoto Prefectural University of Medicine, Kyoto, Japan; Pennsylvania State Hershey College of Medicine, UNITED STATES

## Abstract

The absorption spectrum of light is known to be a “molecular fingerprint” that enables analysis of the molecular type and its amount. It would be useful to measure the absorption spectrum in single cell in order to investigate the cellular status. However, cells are too thin for their absorption spectrum to be measured. In this study, we developed an optical-cavity-enhanced absorption spectroscopic microscopy method for two-dimensional absorption imaging. The light absorption is enhanced by an optical cavity system, which allows the detection of the absorption spectrum with samples having an optical path length as small as 10 μm, at a subcellular spatial resolution. Principal component analysis of various types of cultured mammalian cells indicates absorption-based cellular diversity. Interestingly, this diversity is observed among not only different species but also identical cell types. Furthermore, this microscopy technique allows us to observe frozen sections of tissue samples without any staining and is capable of label-free biopsy. Thus, our microscopy method opens the door for imaging the absorption spectra of biological samples and thereby detecting the individuality of cells.

## Introduction

Our quantitative understanding of cellular function would be aided considerably by the accurate determination of the amounts of molecular components (such as nucleic acids, proteins, and lipid) present in living cells. One method for such quantitation is the measurement of light absorption, which reveals both the molecular amount and type, as in the commonly used assays for protein [1] and nucleic acid [2] concentration. We wondered if it would be possible to apply the same molecular fingerprinting approach to a living cell at a subcellular resolution to quantitatively estimate the molecular concentration and distribution, as this may reveal differences between cells that bulk biochemistry overlooked.

The absorption of light by a sample (optical density (O.D.)) is determined by the classical Lambert-Beer law, i.e., $\text{O}.\text{D}.=-{\text{log}}_{10}\frac{I_{sample}}{I_{blank}}=\epsilon \cdot c\cdot l$, where *I*_*blank*_ and *I*_*sample*_ are the signal intensities of light measured in a blank sample and the subject sample, respectively; *ε* is the molar absorption coefficient (M^-1^cm^-1^); *c* is the concentration (M); and *l* is the length through which the light travels in the sample. The thickness of the average mammalian cell [3] (~10 μm) combined with an O.D. detection limit of 0.01 should determine a minimum concentration threshold detection limit of ~100 μM. This is approximately one order of magnitude too high for practical use, even if (as in this case) the cell is considered to be expressing a chromogenic protein with a large *ε* (~10^5^ M^-1^cm^-1^), as transient transfection typically produces protein in a ~10-μM range. Indeed, although the light absorption measurement of cells has clear potential, few studies have performed an absorption measurement for chromo-proteins in living cells [4,5], possibly because these methods could not measure O.D. values and visible samples.

Considering the Lambert-Beer law equation, *ε* and c are values unique to the molecule of interest and thus cannot be modified. However, we can extend the effective optical pass length *l* if the light can be multiply reflected to pass through the sample many times, allowing multiple absorption events. Optical cavities are capable of this and have, for example, been applied to measure homogeneous samples such as gas mixtures [6] using techniques such as cavity ring-down spectroscopy (CRDS) and cavity enhanced absorption spectroscopies (CEAS) [7–10]. However, these light absorption spectroscopy techniques are one-dimensional, so that application to the biomolecules are limited.

## Results

### Development of cavity-reflection-enhanced absorption microscopy

**Fig 1:**
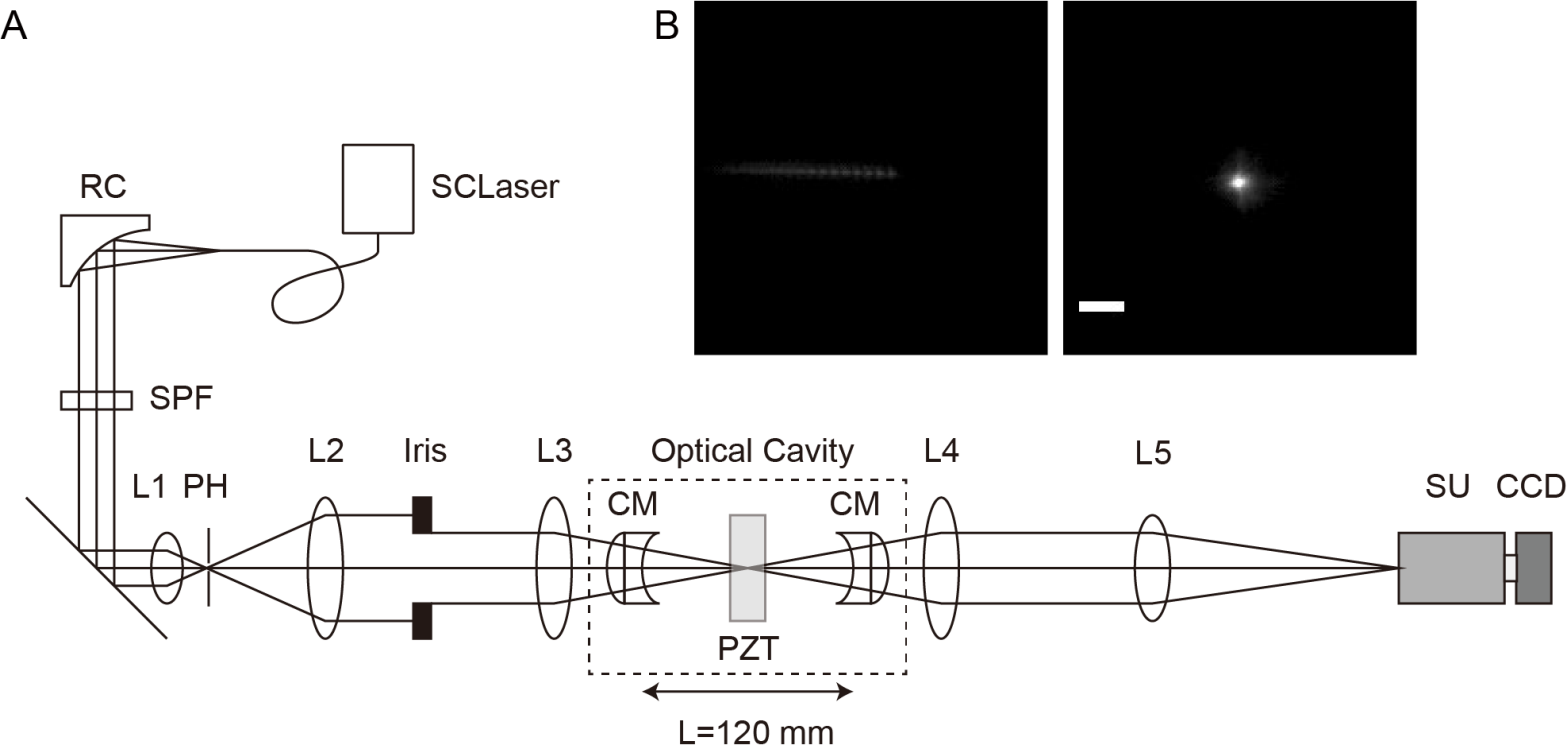
Schematic of cavity-reflection-enhanced absorption microscopy (CREAM). (A) Optical system of CREAM. SCLaser, supercontinuum laser; RC, reflective collimator; SPF, short pass filter; L1 to L5, spherical achromatic lenses; PH, pinhole; CM, cavity mirror; PZT, piezo xy-stage; SU, spectrum unit; CCD, charge coupled-device. Dotted box area indicates optical cavity. We set optical cavity length L = 120 mm for concentric-type optical cavity. (B) Focused spot images of before (left) and after (right) adjustment in the center of cavity measured by CCD camera. Scale bar represents 50 μm.

We developed a cavity-reflection-enhanced absorption microscopy (CREAM) method allowing two-dimensional absorption spectrum imaging (Fig 1A). A spherical concentric optical cavity system [11] was chosen, with the cavity length set to 120 mm using 60-mm curvature concave mirrors with a reflectivity of 99.5% for the wavelength range of 400 to 600 nm (see Materials and Methods). A supercontinuum laser was collimated and focused at the center of the cavity by a spherical achromatic lens passing through the optical cavity mirror. The full width at half maximum of the focused light was 14.17 μm for the x-axis and 15.62 μm for the y-axis (Fig 1B and S1 Fig).

**Fig 2:**
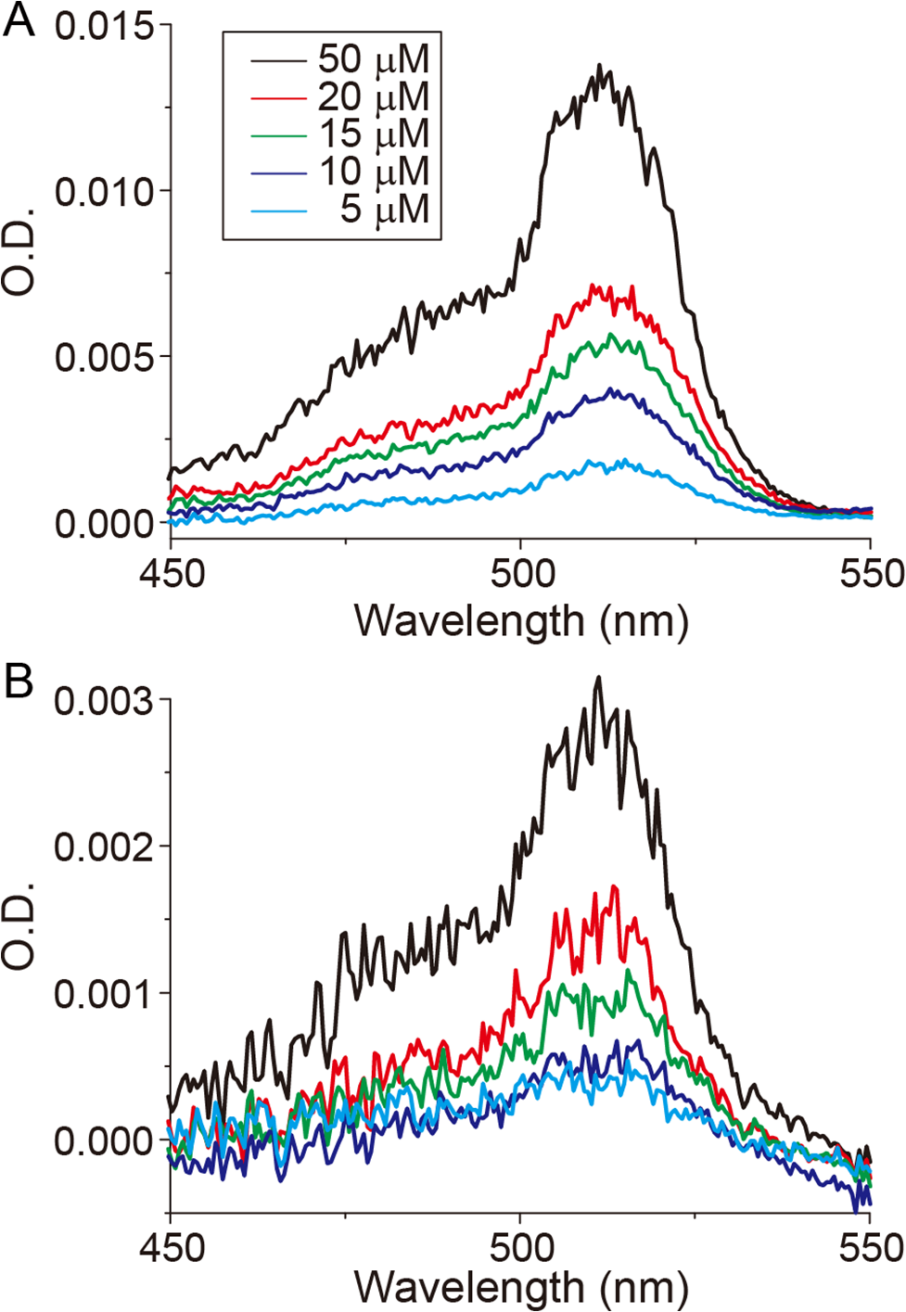
Light-absorption spectrum of Venus yellow fluorescent protein by CREAM. Various concentrations of Venus fluorescent protein with different sample thicknesses of (A) 100-μm and (B) 10-μm.

To verify our homemade CREAM system, we first measured various concentrations of purified recombinant Venus [12] yellow fluorescent protein with different optical path lengths *in vitro*. With 100-μm and 10-μm optical path lengths, we measured the absorption spectrum of the Venus (Fig 2) using the following equation (S1 Text):

$$\text{O}.\text{D}.(\lambda )=0.434\cdot T(\lambda )\cdot \frac{I_{blank}(\lambda )-I_{sample}(\lambda )}{I_{sample}(\lambda )},$$ (1)

where *I*_*sample*_ and *I*_*blank*_ are the light intensities for the actual samples and blank samples such as a buffer, respectively, and *T* is the transparency of the optical cavity mirrors. Because the 10-μm optical path length is 1,000 times smaller than that of a conventional absorption spectrometer using a 10-mm optical-path-length cuvette, the O.D. detection threshold becomes 1,000 times smaller. Indeed, using conventional absorption spectroscopy, for 50-μM Venus (ε is 92,200 M^-1^cm^-1^) [12] with a 10-μm-optical-path-length sample, the estimated O.D. is only 0.00461, which is beyond the detection limit of the conventional system, whereas CREAM can detect an O.D. value of 0.0005.

### Spectral fingerprinting for various types of cells

**Fig 3:**
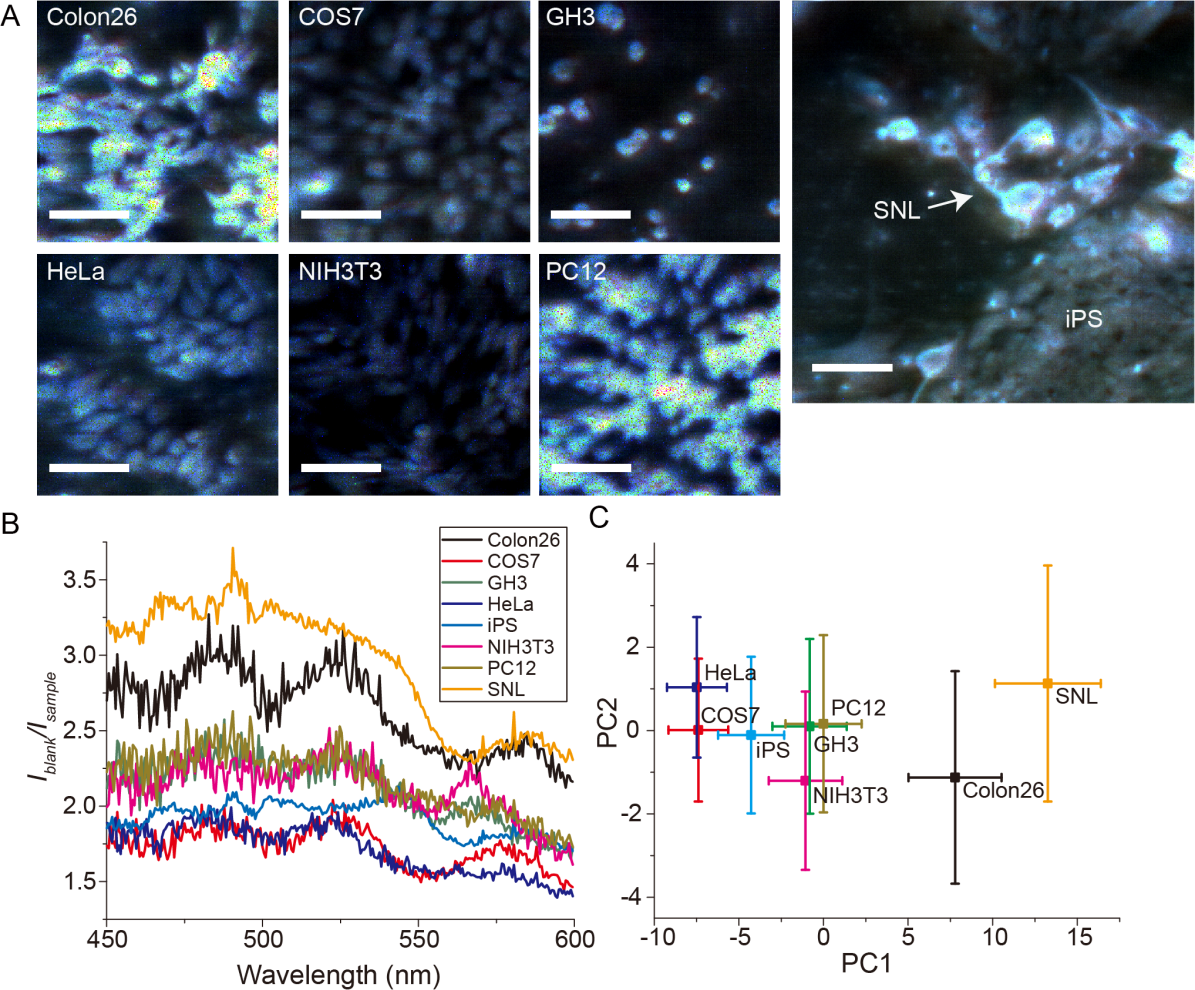
Absorption images and spectra of various cell types. (A) Cavity-reflection-enhanced absorption images for cell types as indicated. Pseudo-colored images were constructed by merging the 3 colors as follows: blue for 450 to 500 nm, green for 500 to 550 nm, and red for 550 to 600 nm of averaged images. Scale bars represent 100 μm. (B) Averaged spectrum of each cell type (n = 10 for each cell type). Colors correspond to the different cell types in Fig 3B. (C) Scatter plot of PCA scores by PC1 and PC2 components. Error bars represent standard deviation.

Because cells are apparently transparent, simple bright-field optical microscopy has little intrinsic contrast within the sample that yields useful image information. Therefore, several techniques, such as phase contrast microscopy, i.e., differential interference microscopy, have been developed in order to enhance the intrinsic contrast of biological samples and improve the image quality [13]. However, these techniques do not provide spectral absorbance information, which is quantitatively related to the molecular composition of the cell. To demonstrate the superiority of CREAM compared with conventional contrast-enhancement methods, we conducted two-dimensional spectral CREAM for various cells types, including HeLa, GH3, PC12, Colon26, COS7, NIH3T3, and human iPS grown on an SNL (mouse) feeder monolayer. The blank signal was taken from the region in the field of view where there is no cell, i.e., only the mounting medium (Gelvatol) were filled. Although high-contrast absorption images were captured for each cell type (Fig 3A and S2A Fig) using CREAM, the removal of the optical mirrors to produce a conventional imaging setup lead to a clear loss of the image contrast (S2B Fig). Compared with the homogenous solutions, cells have heterogeneous structures that cause light scattering and refraction. Eq (1) is valid for homogeneous, low-scattering samples [9]. Therefore, we simply calculated the inverse of the transparency, *I*_*blank*_ / *I*_*sample*_, where *I*_*blank*_ represents the blank signals obtained by the empty region of interest, and *I*_*sample*_ represents the sample signals.

As expected, we discovered that each cell type produced a stereotyped absorption spectral fingerprint in the wavelength range of 450 to 600 nm (Fig 3B). In order to investigate the characteristic features of the spectra, we performed a principal component analysis (PCA), whereby four major principal components (PCs) were extracted (S1 Table). The proportion of the PCs indicated that the 1^st^ PC (PC1) was the main feature. PC1 exhibited an almost constant value with respect to the wavelength, suggesting that the 1^st^ PC score represents the signal intensities of the spectrum. Although the other PC components—PC2, PC3, and PC4—exhibit low proportions compared with PC1, the fluctuated signals corresponded to the peaks of the spectra of cells, suggesting that PC2, PC3, and PC4 indicated the characteristic spectral peaks (S3 Fig). We did not expect this technique to reveal small differences in the absorbance between individual cells of the same cell type, which may reflect the personality of cells or the cellular status (Fig 3C and S4 Fig). Furthermore, we analyzed absorption spectra of HeLa cells and COS7 cells whose cell-cycles were synchronized by double-thymidine block method (S5A Fig and S5B Fig, respectively). The scatter plot of PC1 and PC2 scores of individual HeLa cells and COS7 cells indicated the close but significantly different distributions (S5C Fig), suggesting the cell type dependent absorption spectra. Owing to the small absolute cell thickness [14,15] and the implicit requirement to average the absorbance spectra in a sample, a conventional macroscopic spectrometer cannot detect these subtleties (S6 Fig).

Next, we observed and compared the absorption spectra of COS7 cells transiently expressing Venus fluorescent proteins (S7A Fig). When *I*_*blank*_ / *I*_*sample*_ was calculated by using empty region of the sample as blank signal, Venus absorption spectrum was not observed because of the intrinsic intense signal at around 525 nm (S7B Fig, black line). However, when the blank signal was taken by cell, absorption spectrum peaking at 515 nm was observed (S7B Fig, red line). Because the absorption maximum of the Venus fluorescent protein is 515 nm [12], the observed spectrum attributed Venus absorption.

### Absorption imaging for rectal tumor tissues

**Fig 4:**
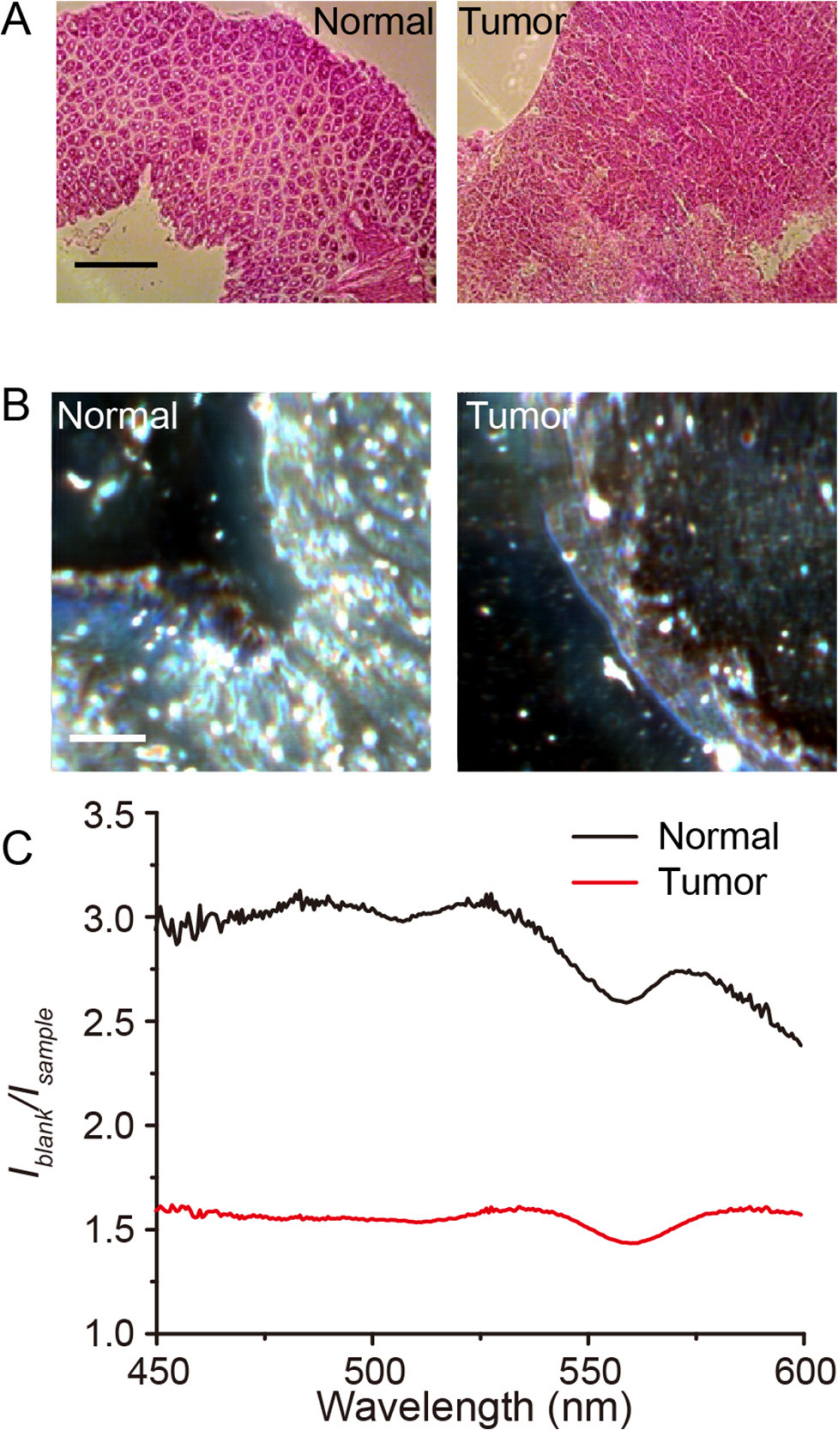
Observation of human rectal tumor model and mouse normal rectal tissues. (A) Hematoxylin-Eosin stained mouse normal rectal tissues (left) and human rectal tumor model (right). Scale bar represents 0.5 mm. (B) Cavity-reflection enhanced absorption images of mouse normal rectal tissues (left) and human rectal tumor model (right). Scale bar represents 100 μm. Pseudo-colored images were constructed by merging the 3 colors as follows: blue for 450 to 500 nm, green for 500 to 550 nm, and red for 550 to 600 nm of averaged images. (C) Cavity-reflection-enhanced absorption spectrum of mouse normal rectal tissues (black) and human rectal tumor model (red).

Because the spectral fingerprint appeared specific to the cell type, we investigated its potential for clinical application, for example, in a malignant process where cell types can become mixed during a local invasion or metastasis. Currently, histological and immunohistochemical staining are the standards in clinical care (Fig 4A), but these techniques require prior knowledge of what the tumor may express, along with processing and observation, all of which are subject to various errors contributing to reductions in the test sensitivity and specificity. In an orthotopic mouse model of human rectal carcinoma, we were able to compare rectal tumor and normal tissues by the reconstruction of pseudo-colored images (Fig 4B). Interestingly, the normal and tumor tissues exhibited significantly different spectra (Fig 4C), suggesting the different amount of the molecular components and tissue structures.

## Discussion

Because the absorption is proportional to the concentration and optical path length, as predicted by the Lambert-Beer law, conventional absorption spectroscopy requires large sample volumes and concentrations. CREAM uses a light focused on a ~15-μm spot in an optical cavity (S1 Fig), with which we can measure the O.D. of a Venus fluorescent protein at an optical path length of 10-μm (Fig 2), to a concentration of approximately 5 μM. These results suggest that CREAM has the potential to measure very small sample volumes. However, the obtained O.D. values in 100-μm and 10-μm optical path length were lower than the expected O.D. values. For example, the O.D. values at 515-nm of 50-μM Venus solution with 100-μm and 10-μm optical path length were expected 0.0461 and 0.00461, respectively, whereas the O.D. values obtained by CREAM with 100-μm and 10-μm cuvette were ~0.015 and ~0.003, respectively. The O.D. values in CREAM derived by the Eq 1 suppose the infinite repetitions inside the optical cavity. However, because of the scattering by sample, the repetition numbers inside the cavity could be decreased, making the substantial optical path length short. Such reduction effect was large in thick cuvette (i.e. 100-μm distance). Because the incident light is focused at the center of cavity, the insertion of the thick sample may disturb the ray of light. Further improvement of the sample chamber for thick samples should be desired. Suppose that the shape of the focused light is a cylindrical column with an average diameter of 14.9 μm, derived from the FWHM of the spot size (Fig 1B), (14.9 μm / 2)^2^ × *π* × 10 μm = 1,743.7 μm^3^ (∼ 1.7 pL), at concentrations beneath the current detection limits. For samples of an even lower concentration, the use of an anti-reflection-coated cuvette may further improve the detection limit. The spatial resolution is determined according to the spot size inside the optical cavity. The current spatial resolution is ~15-μm, which is sufficient for the detection of individual cells. However, we failed to observe sub-cellular structures such as the mitochondria and nucleus, because of the low spatial resolution of low-numerical-aperture optics. In addition, the chromatic aberration may cause spatial resolution degradations. The optimization of CREAM using high-numerical-aperture optics could improve the spatial resolution.

The problem of the phototoxity is one of the major issue for live cell imaging. Like other types of optical microscopy, CREAM uses a light for the measurement of the absorption spectra. However, the light power density inside the cavity mirror was only ~0.3 W/cm^2^ at 550 nm that showed the highest intensity of the incident light. Indeed, we could observe the cell migration of the *dictyostelium discoideum* cells that were easily affected by the phototoxic effect (S1 Video). These results suggest negligible phototoxity of CREAM for live cells.

By combining a scanning stage with grating equipment in front of a CCD, we generated a two-dimensional absorption spectrum image. CREAM greatly increases the image contrast compared to the non-absorption enhanced image (S2A Fig and S2B Fig, respectively). The image contrast can be estimated by the gain that is calculated as the ratio of enhanced to non-enhanced signals as follows, $\text{gain}=\frac{\left(I_{CREAM}-background\right)}{\left(I_{non-CREAM}-background\right)}$ where *I*_*CREAM*_ and *I*_*non-CREAM*_ were the invers of transparencies (i.e., *I*_*blank*_ / *I*_*sample*_). The estimated gain at 525 nm was approximately 206 (S2C Fig).

Interestingly, the spectral patterns were different between the cell types and even among cells of the same type, suggesting the distinct cellular compositions of the absorption substrates. Further research is required to determine the cause of these peaks. Some molecules have well-known absorption spectra; for example, it is known that the flavin mononucleotide has absorption peaks around 450 nm, and cytochrome c has absorption peaks at 520 and 550 nm in the reduced form [16,17]. Our data indicated absorption peaks around these wavelengths, indicating the potential for the observation of the flavin mononucleotide and cytochrome c. However, some cells exhibited different peak positions at these wavelengths and others. For example, the iPS and SNL cells showed a different peak pattern around 570 nm compared with other types of cells in an averaged spectrum (Fig 3B). In addition, some GH3 and colon26 cells exhibited different spectra patterns compared with other cells of the same type (S4 Fig). These results suggest that the composition is cell-type-dependent or cell-condition-dependent. Furthermore, we could distinguish the pattern of absorption spectra of HeLa cells from that of COS7 cells, even if those cellular stages were synchronized to minimize differences within one population. This result suggests that CREAM can be distinguish cell species.

The PCA scores also indicated cell type similarities and differences (Fig 3C and S3 Fig). According to the scatter plot indicating the PC1 and PC2 scores (Fig 3C), the pairs of HeLa and COS7 cells, PC12, GH3, and NIH3T3 exhibited a similarity, whereas Colon26, SNL, and iPS exhibited different PC scores. The coefficient of PC1 exhibited an almost constant value for the entire wavelength range, indicating that PC1 reflects the intensity of the spectrum itself. On the other hand, PC2, PC3, and PC4 exhibited characteristic positive or negative peaks corresponding to the cellular spectra, suggesting that these PC2, PC3, and PC4s indicate the spectrum information, despite the low proportions (S1 Table).

The fluorescence microscopy such as confocal and multi-photon fluorescence lifetime imaging microscopy can also detect the autofluorescence with high sensitivity [18,19]. Spectral unmixing method can clearly isolate the autofluorescence. Laser-scanning type fluorescence microscopies can focus the light at the specimen with diffraction limit size (~200 nm) and thus the volume size is fL order, which is 1,000 times smaller than that of the CREAM. In spite of the high sensitivity, the fluorescence microscopy cannot detect light absorption at all. In contrast, CREAM can detect the light absorption by the samples such as chromoproteins and chromogenic dyes that do not emit fluorescence. Thus, CREAM can be useful as an alternative measurement method.

Because light passing through a sample contains information about the light absorption and the light scattering, conventional cuvette spectrometry measures a blank sample to eliminate the light-scatting effect. However, in CREAM for cellular imaging, blank measurements are made in cell-deficient regions, and thus the light-scattering effect may remain in CREAM measurements. In addition, for sample measurement by CREAM, we used two different expressions: O.D., which is used for the Venus solution measurement, and *I*_*blank*_ / *I*_*sample*_. The former is compatible with the values of conventional spectrometers. In contrast, the latter is not O.D. but rather the inverse of the transparency of the sample. Because the cells are not homogeneous and have some scattering (S2 Fig), actual O.D. values cannot be obtained. The Venus absorption spectrum was measured in the cell (S7 Fig). However, due to the difficulty to obtain the O.D. value inside the cell as described above, the estimation of the Venus concentration in the cell could not be achieved. In addition, signals derived from the “auto-absorption” hinder the Venus signals. Further study should be required to obtain the O.D. value inside the cells. However, as the differences in the cell type and individual cell spectrum are attributable to fundamental differences in the cellular composition under equivalent experimental conditions, CREAM may be useful not only for the investigation of the cellular individuality but also for innovative drug development and quantitative pathological diagnosis.

## Materials and Methods

### Ethics Statement

All animal experiments were conducted with approval and in accordance with guidelines from the Committee for Animal Research, Kyoto Prefectural University of Medicine (Permit Number: M25-41).

### Optical system

The output of a supercontinuum laser (SM-30, LEKOS) from an optical fiber was collimated by a parabolic mirror (EC12SMA-F01, Thorlabs, Inc.). The collimated beam was reflected by a dichroic mirror (FF593-Di02-25x36, Semrock, Inc.) to separate the visible and infrared light and passed through a short-pass filter (FES0600, Thorlabs, Inc.) to reduce the infrared light as much as possible. The collimated light beam was expanded by a Galileo-type expander (DLB-25-40PM and DLB-50-120PM, Sigma Koki Co., LTD.) with a 5-μm pinhole (PH-5, Sigma Koki Co., LTD.) for spatial filtering. The beam size was adjusted to 24 mm by the iris and focused by the spherical achromatic lens (DLB-50-120PM, Sigma Koki Co., LTD.). The focused light entered 60-mm curvature, 99.5% reflectivity, 0.5% transparency, and the 400-to-600-nm-wavelength optical cavity mirror (LCM99.5Q-60-400-600, Sigma Koki Co., LTD.). Because the cavity mirror is a plane-concave mirror, the focused light path across its plane surface is not straight but is bent by refraction, causing spherical aberration. To solve this problem, we attached a customized plane-convex lens (SLSQ-25tc4-150P, Sigma Koki Co., LTD.) in order to produce a straight light path. Because the concentric-type optical cavity is unstable, we used a kinematic piezo mirror mount (PZ-631B/M, Thorlabs, Inc.) for fine optical light angle adjustment and a one-axis piezo stage (SFS-H60X (XL), Sigma Koki Co., LTD.) for fine optical cavity mirror length (120 mm) adjustment. For the adjustment, multiple reflected spots were observed and converged to one spot by the adjustment mirror angle, and then optical cavity mirror length was adjusted in order to maximize the output light intensity. After exiting the optical cavity, the light was collimated by a spherical achromatic lens (DLB-50-120PM, Sigma Koki Co., LTD.) and focused spherical achromatic lens (DLB-30-300PM, Sigma Koki Co., LTD.) to the detector. For the spectrum measurement, a spectrometer (CLP-50, Andor Technology) was coupled to an EMCCD (Luca-S, Andor Technology) in fast external triggered multi-track mode using image acquisition software (Solis, Andor Technology) with a 500-μs to 2.5-ms exposure time. The samples were held to the custom-made long travel distance (~ 1 mm) piezo xy-stage (Chuo Precision Industrial Co., LTD) in the center of the optical cavity and moved by 1-μm steps in the xy-direction over a 5-ms duration. Stage control was performed using homemade software in LabView (National Instruments Corp.) and synchronized with spectrum detection by a CCD camera using a data acquisition device (NI PCIe-6321, National Instruments Corp.). The spectrum image data were also analyzed using homemade software in LabView and ImageJ.

### Protein purification and preparation of Venus

An *E coli* strain JM109 (DE3) was transformed with a pRSET_B_ vector carrying Venus fluorescent protein and grown at 23°C. The recombinant protein was purified using Ni-NTA agarose (Qiagen) and diluted with PBS (Takara bio, Inc.). Various concentrations of the Venus protein (5, 10, 20, and 50 μM) were adjusted by measurement using an absorption spectrometer (V-630BIO, Jasco Corp). The Venus was inserted into a glass cuvette made of a cover glass (No. 1S, Matsunami glass) with 100-μm or 10-μm-thick double-sided tape (No.5610 and No.5601, respectively, Nitto Denko Corp.) and sealed using Vaseline (Wako Pure Chemical Industries, Ltd.).

### Cell culture and fixation

HeLa, COS7, and NIH3T3 cells (RIKEN BRC) were cultured with a Dulbecco Modified Eagle medium (DMEM, D6046, Sigma Aldrich Co. LLC.) and 10% Fetal Bovine Serum (FBS, BioWest) on a collagen (Cell Matrix Type 1-C, Nitta gelatin Inc.)-coated cover glass (No.1S grade, Matsunami Glass Ind., LTD.) in a 5% CO_2_ incubator at 37°C. Human iPS cells (201B7, RIKEN BRC) were cultured with a primate ES medium (Reprocell Inc.) and a 4-ng/mL human basic fibroblast growth factor (Wako Pure Chemical Industries, Ltd.) in a 5% CO_2_ incubator at 37°C on a mouse feeder cell layer (SNL, CBA-316, Cell Biolabs, Inc.). PC12 cells (RIKEN BRC) were cultured with DMEM containing 10% horse serum (HS, 16050–122, Life technologies) and 5% FBS on a collagen-coated cover glass (No.1S grade, Matsunami Glass Ind., LTD.) in a 10% CO_2_ incubator at 37°C. A 100-ng/mL nerve growth factor (G5141, Promega KK.) was added to induce differentiation for 3 days. The GH3 cells (JCRB Cell Bank) were cultured with DMEM containing 15% HS and 2.5% FBS on a poly-D-lysin (P7886-60MG, Sigma Aldrich)-coated cover glass (No. 1S grade, Matsunami Glass Ind., LTD.) in a 5% CO_2_ incubator at 37°C. Colon26 (RIKEN BRC) cells were cultured as described previously [18].

For fixation, the cells were washed by PBS twice and then incubated with a DMEM/F12 medium (11039–21, Life Technologies) for 20 min in a 5% CO_2_ incubator at 37°C. Next, they were treated with 4% formaldehyde for 10 min at room temperature and then washed by PBS. Subsequently, the PBS was removed and dried. Then, 5-μL Gelvatol [19] was applied and covered by a cover glass (No. 1S grade, Matsunami Glass Ind., LTD.) with 10-μm-thick double-sided tape (No.5601, Nitto Denko Corp.).

### Cell-cycle synchronization

The cell-cycle of HeLa cells and COS7 cells were synchronized by using double thymidine block method [20]. Cells were plated (2×10^4^ cells) with a culture medium of DMEM and 10% FBS in each of 6 wells plate (353046, Corning) with a collagen coated cover glass for 24 hours in a 5% CO_2_ incubator at 37°C. The medium was replaced with 3.5 mM thymidine (Wako Pure Chemical Industries, Ltd.) and incubated for 24 hours. After that, medium was replaced and incubated for 18 hours. Next, the medium was replaced with 3.5 mM thymidine again and incubated for 24 hours. Then, the medium was replaced and incubated for 10 hours. This treatment increase the population of the cell-cycle at G2/M phase. Finally, cells were fixed as described above.

### Transfection of Venus expressing vector in COS7 cells

COS7 cells were transfected with pcDNA3 vector carrying Venus fluorescent protein by Lipofectamine 2000 (Life technologies) following product protocols. Cells were fixed as described above.

### Preparation of *Dictyostelium discoideum*

The wild type of *Dictyostelium discoideum* cells (AX-2, Dicty Stock Center) were cultured and starved as described previously [21]. After 5 hours starvation, cells were suspended and immersed into a glass cuvette made of a cover glass (No. 1S, Matsunami glass) with 100-μm-thick double-sided tape (No.5610, Nitto Denko Corp.) and sealed using Vaseline (Wako Pure Chemical Industries, Ltd.). Time-lapse images were taken every 10 min. at 22°C.

### PCA of cell spectrum

For the PCA calculation, we used the averaged spectra of the cells as the input data. The PCA was performed using a princomp function of the R software. The PCA score was calculated using the following equation: *Z*_*m⋅k*_ = *X*_*m⋅k*_ ± *s*.*d*. × *A*_*n⋅k*_, where *Z* is the PCA score, *X* is the averaged spectral data with the standard deviation (*s*.*d*.), *m* is the number of cells, *A* is PC, *n* is a data number of the spectra, and *k* represents the contracted data obtained by the PCA.

### Absorption measurement of HeLa cells by spectrometer

HeLa cells were grown as described above. The cells were harvested and washed with 10 mM Tris-HCl (pH 7.5) twice. Then, 10^6^ cells/ml were resuspended in 10 mM Tris-HCl (pH 7.5), 2 mM MgCl_2_, and 0.5% Triton X-100 and homogenized by extrusion using a 23 gauge needle. The samples were then centrifuged at 1,000 r.p.m. for 10 min. The supernatant light-absorption spectrum was measured using a spectrometer (V-630BIO, Jasco Corp).

### Frozen section of human rectal tumor model and mouse normal rectal tissues

An orthotopic mouse model of human rectal carcinoma was prepared as follows. A BALB/c nude mouse (5-week-old, female) was anesthetized with pentobarbital sodium (40 mg/kg body weight, intraperitoneal), and the anorectal wall was cut to prevent colonic obstruction resulting from the rectal tumor progression. HT-29 cells (5×10^6^ total), cultured with McCoy’s medium (12330–031, Gibco) containing 10% FBS (SH30910.03, Thermo Fisher Scientific) and 1% penicillin streptomycin (15140–122, Gibco) in a 5% CO_2_ incubator at 37°C, were injected submucosally into the rectum of the mouse. After the surgery, tumor size was monitored throughout the tumor progression daily. If the tumor size reached to 17 mm in diameter, animals were sacrificed with an overdose of pentobarbital sodium (200 mg/kg body weight, intraperitoneal) as humane endpoint. After 6 weeks, the rectal tumor and normal rectal tissue were excised from the mouse after euthanasia with an overdose of pentobarbital sodium (200 mg/kg body weight, intraperitoneal). The tumor and the normal tissue were frozen and embedded in O.C.T. The frozen sections were sliced to a thickness of 10-μm by a cryostat (CM1900, Leica) and attached to a cover glass (No. 1 grade, Matsunami Glass Ind., LTD.). The samples were embedded into Gelvatol and covered by a cover glass with 10-μm-thick double-sided tape.

## Supporting Information

S1 Fig Line profiles of x- (left) and y-(right) axis of focused spot light at the center of optical cavity. Red solid lines indicate the fitting curve of Lorentz function (S1 Text).
(TIF)

S2 Fig Comparison of enhanced and non-enhanced absorption image of HeLa cells. (A) Cavity reflection enhanced absorption image of HeLa cells. Scale bar represents 100 μm. (B) Non-cavity enhanced absorption image of HeLa cells in the same field of view shown in (A) Scale bar represents 100-μm. For the measurement of this image, we removed the right side of the optical cavity mirror shown in Fig 1A from optical system. Therefore, this image appears as if taken by conventional bright field microscopy. Pseudo-colored images in (A) and (B) were constructed by merging the 3 colors as follows: blue for 450 to 500 nm, green for 500 to 550 nm, and red for 550 to 600 nm of averaged images. Lookup table of both (A) and (B) images were same. (C) Average spectra of the optical cavity enhanced (CREAM) and non-cavity enhanced (non-CREAM) HeLa cells (n = 28).
(TIF)

S3 Fig Coefficients of principal components analysis data and PCA score plots. (A) Coefficients of PC1 to PC4 with respect to the wavelength. (B) 3-dimensional PCA score plots of PC1, PC3, and PC4 combinations. (C) 3-dimensional PCA score plots of PC2, PC3, and PC4 combinations.
(TIF)

S4 Fig Individual cell spectra of different cell types. Different colors correspond to different cells. Even for the same cell type, the subtle difference in spectral patterns can be seen, reflecting individual cell identity.
(TIF)

S5 Fig Comparison of cell-cycle synchronized HeLa and COS7 cells. CREAM images of HeLa cells (A) and COS7 cells (B). Scale bars represent 100-μm. Pseudo-colored images were constructed by merging the 3 colors as follows: blue for 450 to 500 nm, green for 500 to 550 nm, and red for 550 to 600 nm of averaged images. (C) Scatter plot of PCA scores by PC1 and PC2 components. Each plot indicates the single cell. The distributions of the PC1 scores of HeLa cells (n = 143) and COS7 cells (n = 147), and PC2 scores of those cells were significantly different (Kolmogorov-Smirnov test, *p* = 0.01, OriginPro 9.2).
(TIF)

S6 Fig Absorption spectrum of HeLa cells measured by conventional spectrometer. Lysed HeLa cells with various cellular densities, such as 10^4^ (black), 10^5^ (red), and 10^6^ (blue) cells/ml, as determined by conventional spectrometer.
(TIF)

S7 Fig Cavity reflection enhanced-absorption measurement of COS7 cells that express Venus fluorescent protein. (A) Cavity reflection enhanced absorption image of COS7 that express Venus fluorescent protein. Scale bar represents 100-μm. Pseudo-colored images were constructed by merging the 3 colors as follows: blue for 450 to 500 nm, green for 500 to 550 nm, and red for 550 to 600 nm of averaged images. (B) Absorption spectra of COS7 expressing Venus fluorescent protein by taking the cell as blank (red line) and by taking the empty region as blank (black line). Both signals were taken by the same cell.
(TIF)

S8 Fig Schematic of light-absorption process by optical cavity system. (A) The first light-absorption process. The incident light (*I*_*0*_) passes through the left side of the optical cavity mirror with a transparency *T*, and the passed light is absorbed by the sample with absorbance *A*. Next, the small fraction of light passes through the right side of the cavity mirror. (B) Most of the light is reflected by the right side of the cavity mirror and re-absorbed by the sample. (C) The light is reflected by the left side of the cavity mirror and reabsorbed by the sample. Then, the light passes through the right side of the cavity mirror. (D) The light that passes through the right side of the cavity mirror at the k^th^ time is indicated.
(TIF)

S1 Video Time-lapse of the movement of *Dictyostelium discoideum*. Time-lapse of the *dictyostelium discoideum* in starvation stage were taken every 10 min after 5 hours starvation. The timestamp is indicated in hours:mins. Scale bar represents 100-μm. Pseudo-colored images were constructed by merging the 3 colors as follows: blue for 450 to 500 nm, green for 500 to 550 nm, and red for 550 to 600 nm of averaged images.
(AVI)

S1 Table Importance of principal components. (PDF)

S1 Text Supporting methods. (PDF)
