# Supplementary figures and images for: Spectral Fingerprinting of Individual Cells Visualized by Cavity-Reflection-Enhanced Light-Absorption Microscopy
Source: PLoS One. 2015 May 7;10(5):e0125733. doi: 10.1371/journal.pone.0125733 (PMC4423951; doi:10.1371/journal.pone.0125733)

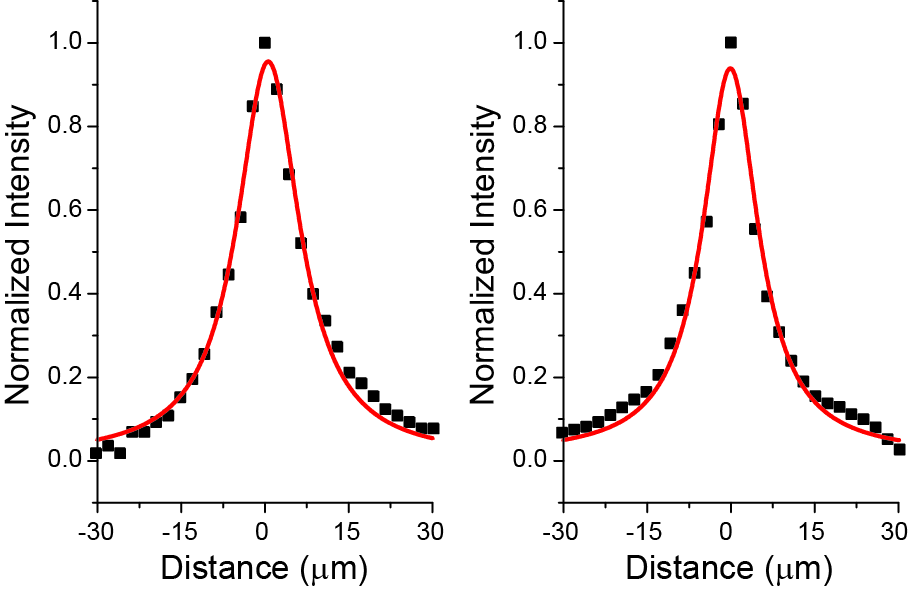

Supplement: S1 Fig — Red solid lines indicate the fitting curve of Lorentz function (S1 Text). (TIF) [file pone.0125733.s001.tif]

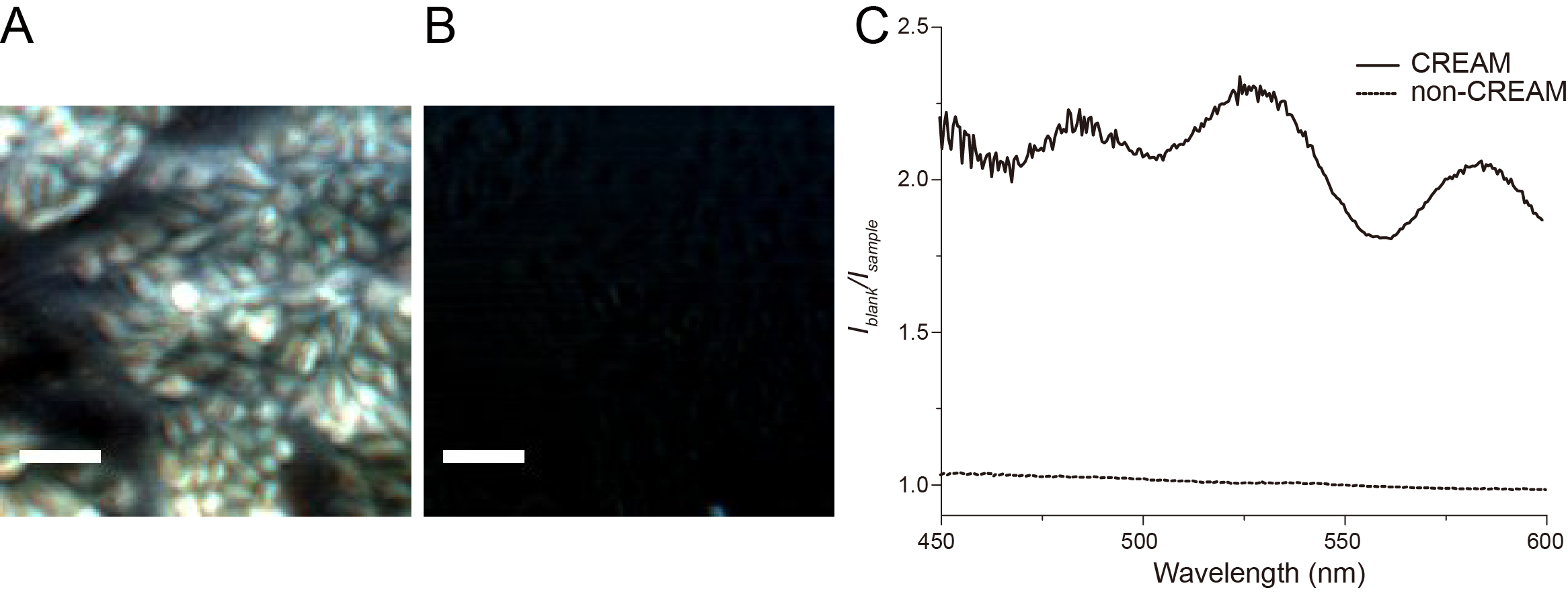

Supplement: S2 Fig — (A) Cavity reflection enhanced absorption image of HeLa cells. Scale bar represents 100 μm. (B) Non-cavity enhanced absorption image of HeLa cells in the same field of view shown in (A) Scale bar represents 100-μm. For the measurement of this image, we removed the right side of the optical cavity mirror shown in Fig 1A from optical system. Therefore, this image appears as if taken by conventional bright field microscopy. Pseudo-colored images in (A) and (B) were constructed by merging the 3 colors as follows: blue for 450 to 500 nm, green for 500 to 550 nm, and red for 550 to 600 nm of averaged images. Lookup table of both (A) and (B) images were same. (C) Average spectra of the optical cavity enhanced (CREAM) and non-cavity enhanced (non-CREAM) HeLa cells (n = 28). (TIF) [file pone.0125733.s002.tif]

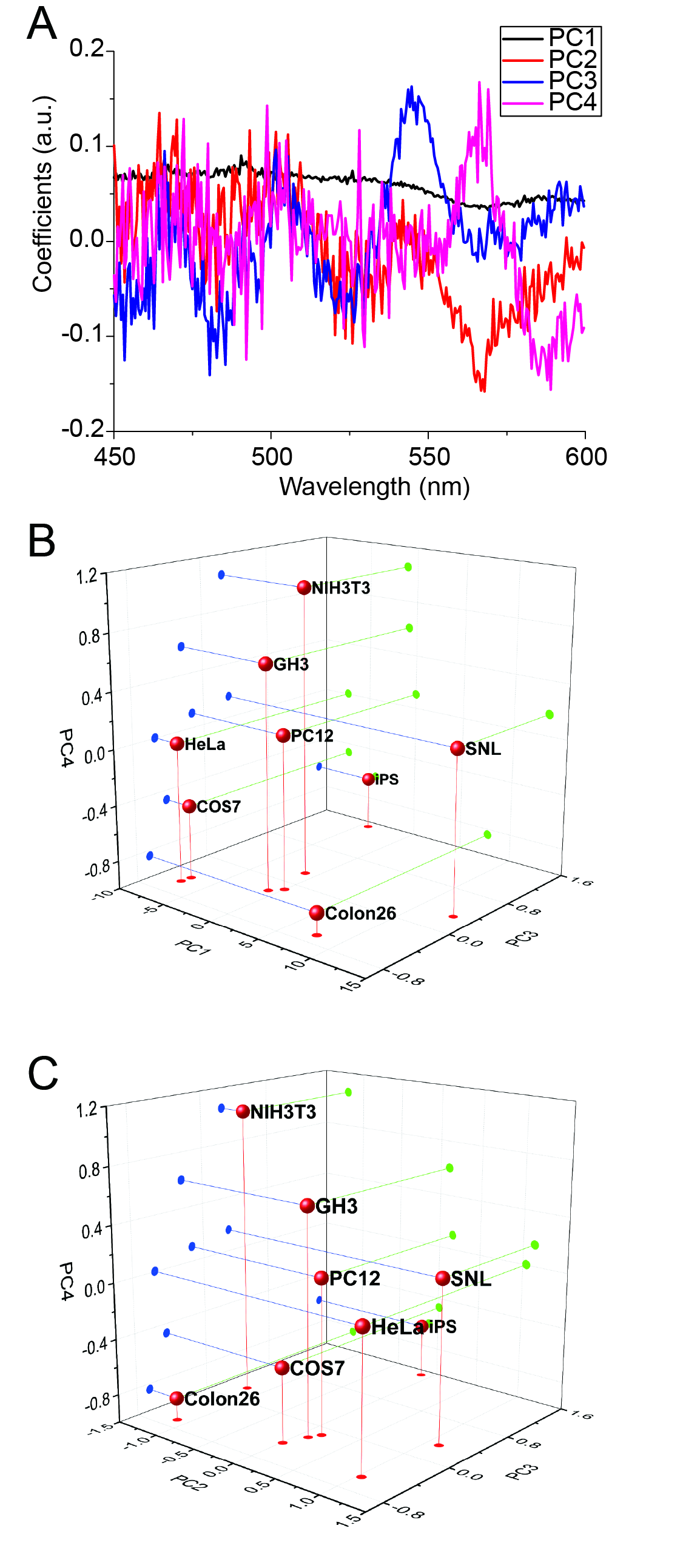

Supplement: S3 Fig — (A) Coefficients of PC1 to PC4 with respect to the wavelength. (B) 3-dimensional PCA score plots of PC1, PC3, and PC4 combinations. (C) 3-dimensional PCA score plots of PC2, PC3, and PC4 combinations. (TIF) [file pone.0125733.s003.tif]

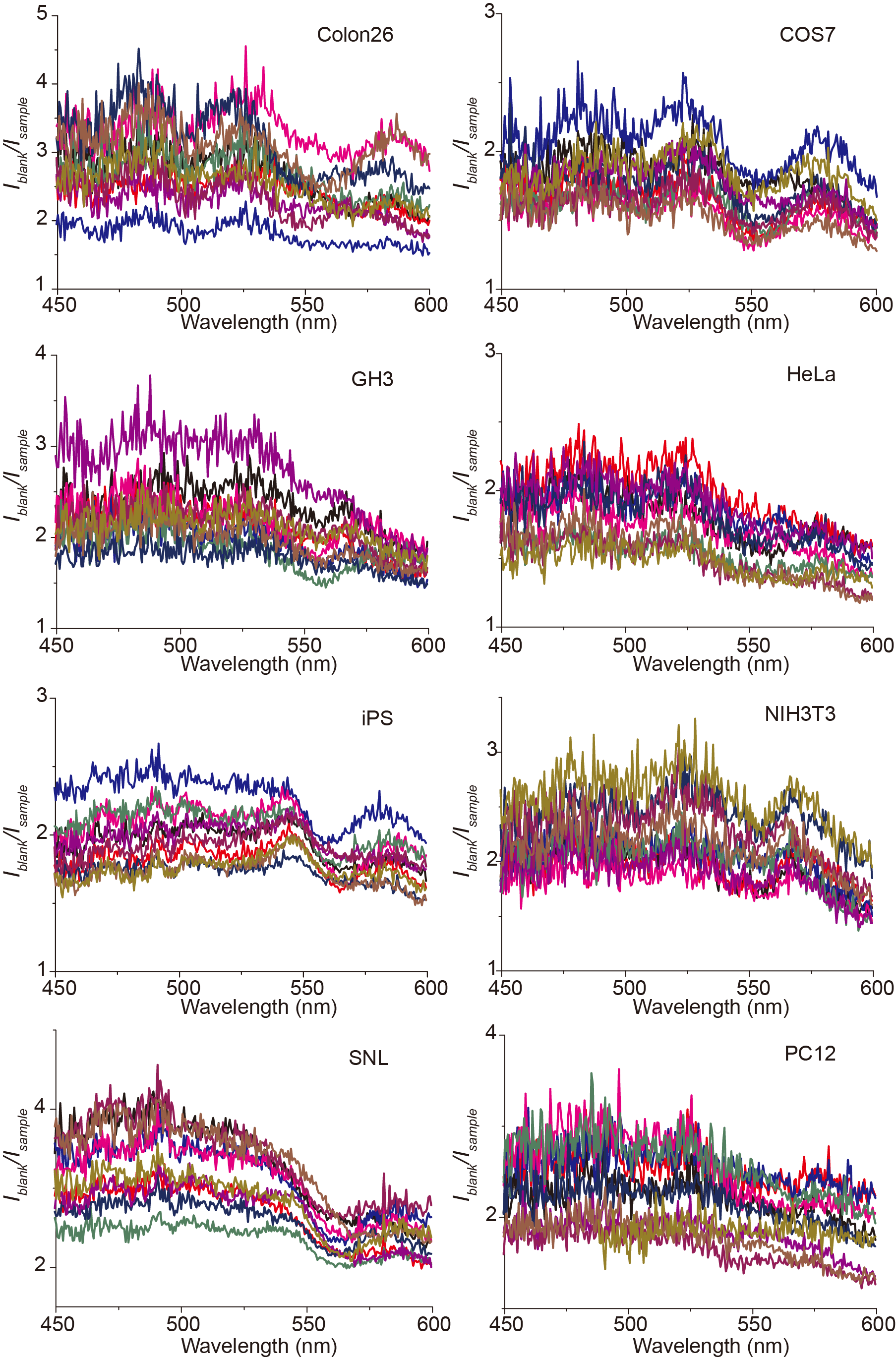

Supplement: S4 Fig — Different colors correspond to different cells. Even for the same cell type, the subtle difference in spectral patterns can be seen, reflecting individual cell identity. (TIF) [file pone.0125733.s004.tif]

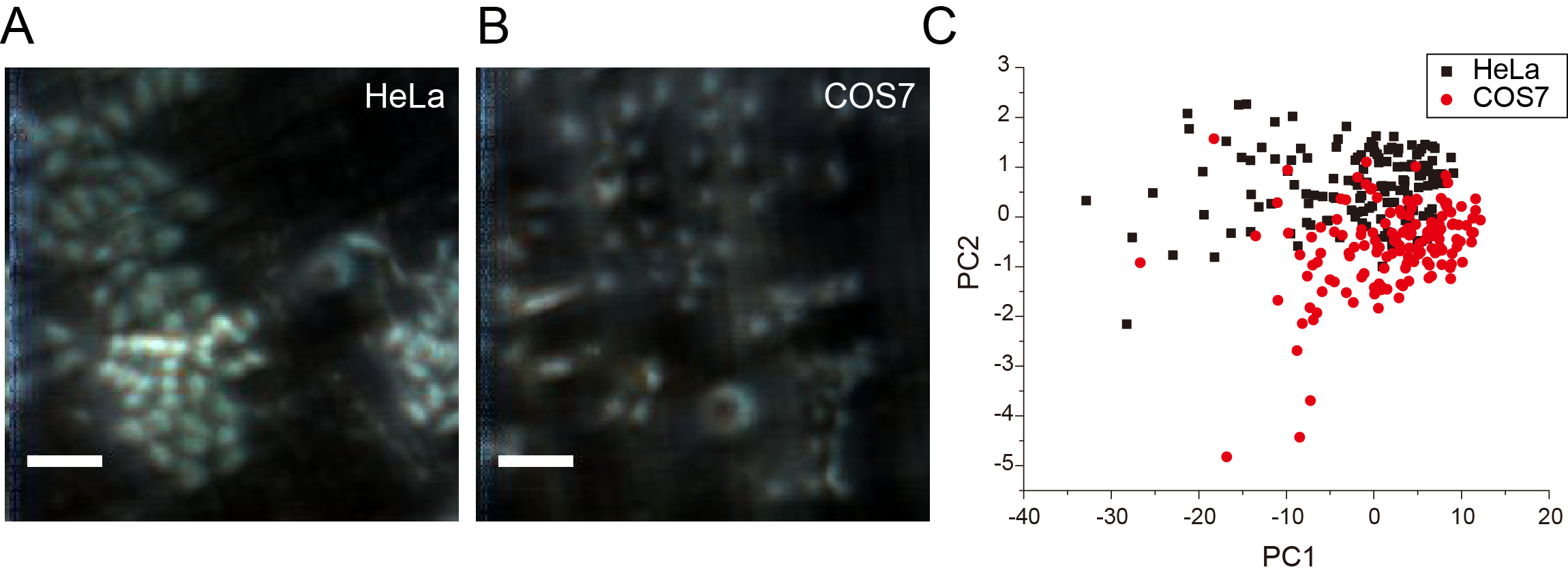

Supplement: S5 Fig — CREAM images of HeLa cells (A) and COS7 cells (B). Scale bars represent 100-μm. Pseudo-colored images were constructed by merging the 3 colors as follows: blue for 450 to 500 nm, green for 500 to 550 nm, and red for 550 to 600 nm of averaged images. (C) Scatter plot of PCA scores by PC1 and PC2 components. Each plot indicates the single cell. The distributions of the PC1 scores of HeLa cells (n = 143) and COS7 cells (n = 147), and PC2 scores of those cells were significantly different (Kolmogorov-Smirnov test, p = 0.01, OriginPro 9.2). (TIF) [file pone.0125733.s005.tif]

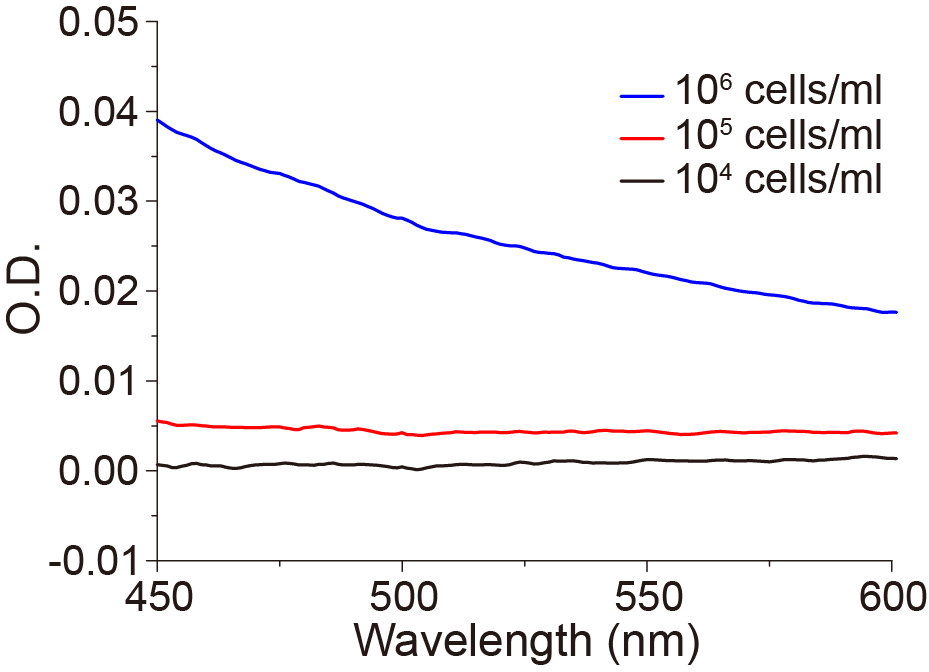

Supplement: S6 Fig — Lysed HeLa cells with various cellular densities, such as 104 (black), 105 (red), and 106 (blue) cells/ml, as determined by conventional spectrometer. (TIF) [file pone.0125733.s006.tif]

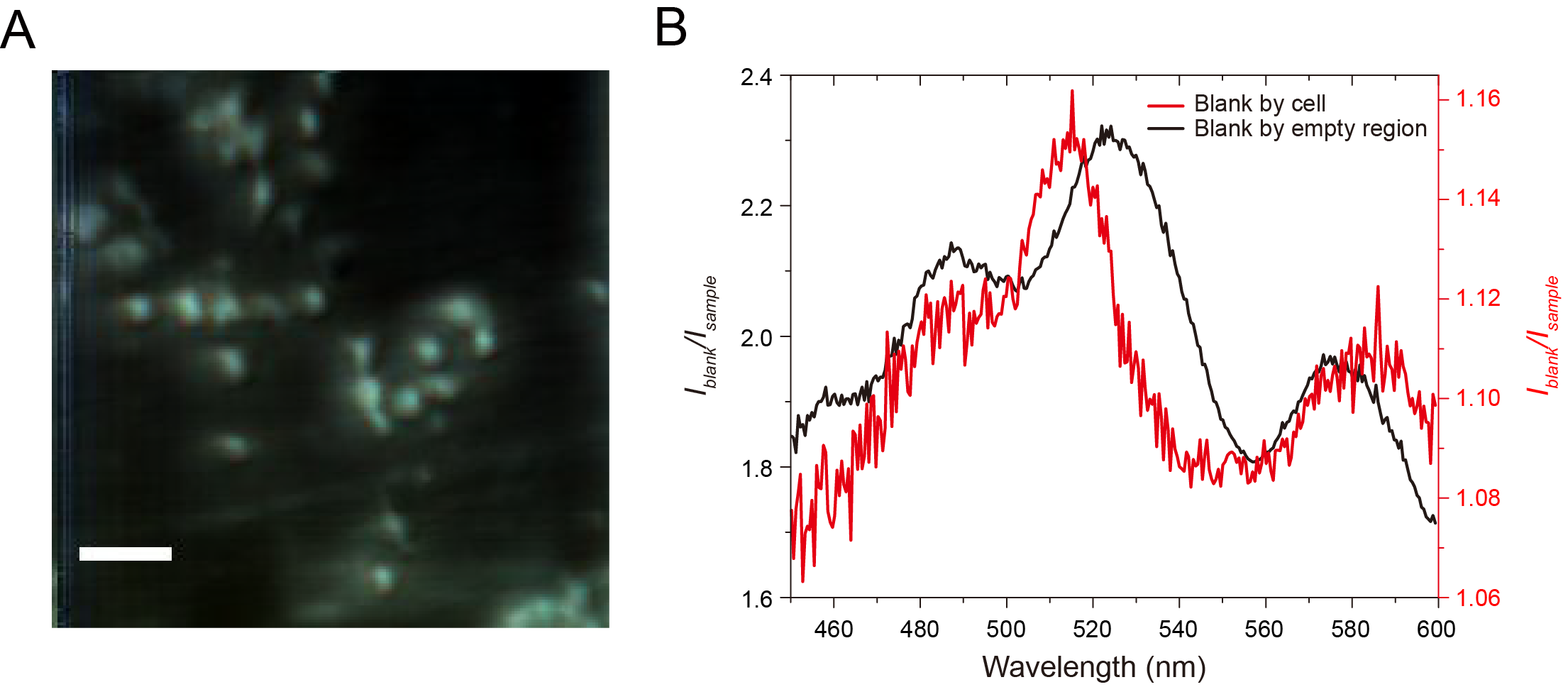

Supplement: S7 Fig — (A) Cavity reflection enhanced absorption image of COS7 that express Venus fluorescent protein. Scale bar represents 100-μm. Pseudo-colored images were constructed by merging the 3 colors as follows: blue for 450 to 500 nm, green for 500 to 550 nm, and red for 550 to 600 nm of averaged images. (B) Absorption spectra of COS7 expressing Venus fluorescent protein by taking the cell as blank (red line) and by taking the empty region as blank (black line). Both signals were taken by the same cell. (TIF) [file pone.0125733.s007.tif]

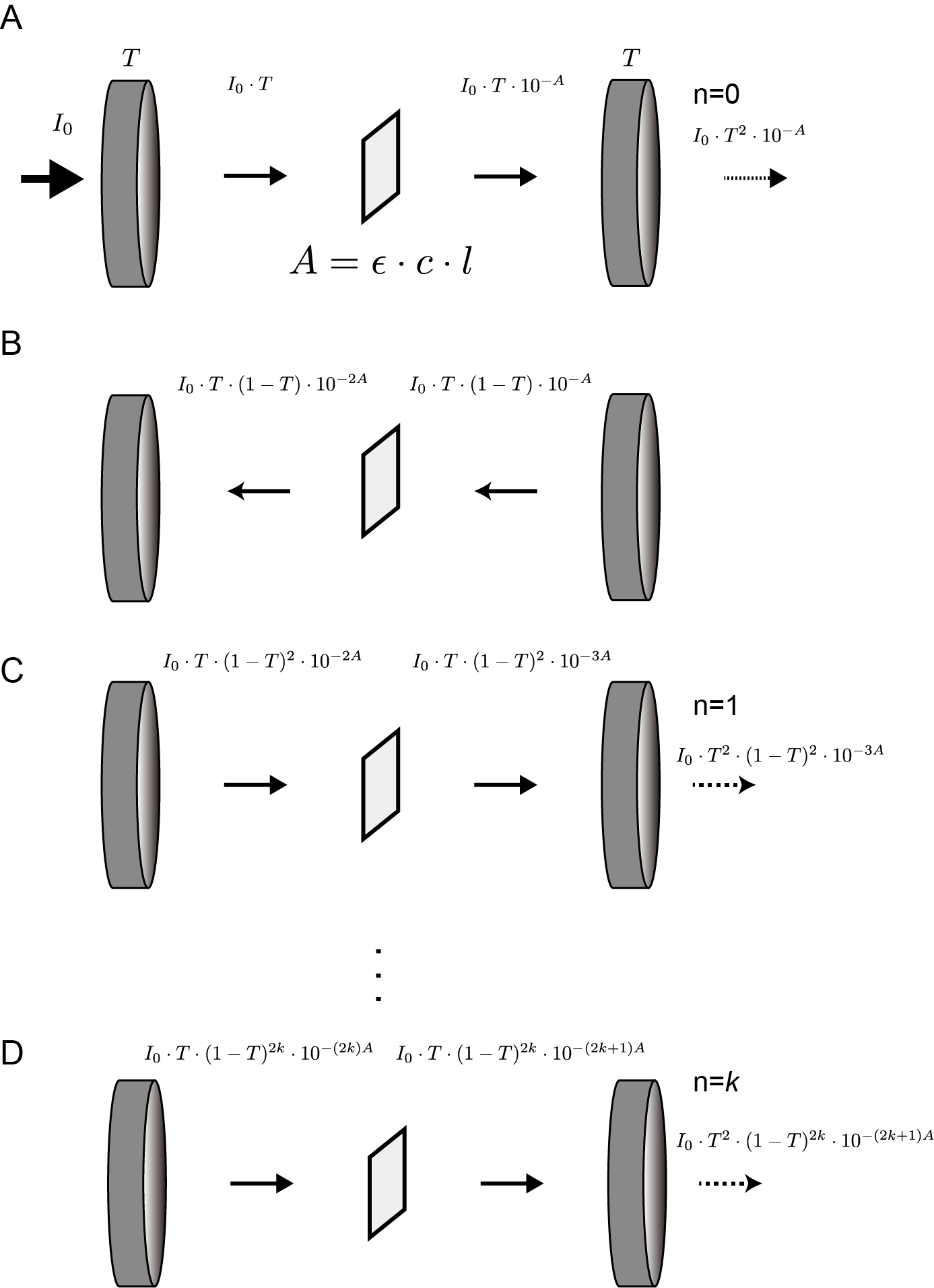

Supplement: S8 Fig — (A) The first light-absorption process. The incident light (I 0) passes through the left side of the optical cavity mirror with a transparency T, and the passed light is absorbed by the sample with absorbance A. Next, the small fraction of light passes through the right side of the cavity mirror. (B) Most of the light is reflected by the right side of the cavity mirror and re-absorbed by the sample. (C) The light is reflected by the left side of the cavity mirror and reabsorbed by the sample. Then, the light passes through the right side of the cavity mirror. (D) The light that passes through the right side of the cavity mirror at the kth time is indicated. (TIF) [file pone.0125733.s008.tif]
