# Supplementary material for: Spectral Fingerprinting of Individual Cells Visualized by Cavity-Reflection-Enhanced Light-Absorption Microscopy
Source: PLoS One. 2015 May 7;10(5):e0125733. doi: 10.1371/journal.pone.0125733 (PMC4423951; doi:10.1371/journal.pone.0125733)
Supplement: S1 Table — (PDF) [file pone.0125733.s010.pdf]

Table S1. Importance of principal components

Principal component	PC1	PC2	PC3	PC4
Proportion of variance	0.9608	0.01341	0.00893	0.00738
