# Supplementary material for: Spectral Fingerprinting of Individual Cells Visualized by Cavity-Reflection-Enhanced Light-Absorption Microscopy
Source: PLoS One. 2015 May 7;10(5):e0125733. doi: 10.1371/journal.pone.0125733 (PMC4423951; doi:10.1371/journal.pone.0125733)
Supplement: S1 Text — (PDF) [file pone.0125733.s011.pdf]

Supporting text

Peak fitting for focused spots

The x- and y-axis line profiles of focused light was well fitted by Lorenz function, $y = y_0 + \frac{2A}{\pi} \cdot \frac{w}{4(x-x_c)^2 + w^2}$, where y_0 was an offset, A was an area of profile, x_c was a center position and w was a FWHM. Fitting was done by Origin9.1 (OriginLab corp).

Calculation of CREAM signals

The enhanced absorption signal is calculated as follows. Suppose I_0 is an incident light, T is an transparency of cavity mirror, A is an absorption of sample (i.e. $A = \epsilon \cdot c \cdot l$), when the incident light propagates from the left side of the optical cavity system, the incident light intensity is dramatically decreased by the left side of the cavity mirror with transparency T. The light intensity is

$$I_0 \cdot T \tag{1}$$

. The light is absorbed by samples, thereby the light intensity is

$$I_0 \cdot T \cdot 10^{-A} \tag{2}$$

The light passes through the right side of the cavity mirror, thereby the light intensity on the right side of the cavity system at first time is

$$I_0 \cdot T^2 \cdot 10^{-A} \tag{3}$$

(S8A Fig.). The most of the light after “first” pass through the sample (eq. 2) is reflected

by the right side of cavity mirror, thereby the light intensity is

$$I_0 \cdot T \cdot (1-T) \cdot 10^{-A} \tag{4}$$

and is absorbed by samples

$$I_0 \cdot T \cdot (1-T) \cdot 10^{-2A} \tag{5}$$

The small fraction of light passes through the left side of cavity mirror (S8B Fig.). Because we detect the light on the right side of the cavity system (Fig. 1A), we ignore the light, which passes through on the left side of cavity.

The light, which is reflected by the left side of cavity mirror, is given by

$$I_0 \cdot T \cdot (1-T)^2 \cdot 10^{-2A} \tag{6}$$

,thereby the light is absorbed by the sample

$$I_0 \cdot T \cdot (1-T)^2 \cdot 10^{-3A} \tag{7}$$

The light intensity which passes through the right side of the cavity mirror is given by

$$I_0 \cdot T^2 \cdot (1-T)^2 \cdot 10^{-3A} \tag{8}$$

(S8C Fig.).

The kth times light intensity which passes through the right side of the cavity mirror is given by

$$I_0 \cdot T^2 \cdot (1-T)^{2k} \cdot 10^{-(2k+1)A} \tag{9}$$

(S8D Fig.). The total light intensity which passes through the right side of cavity is given by

$$\sum_{k=0}^{\infty} I_0 \cdot T^2 \cdot 10^{-A} \cdot \left\{(1-T)^2 \cdot 10^{-2A}\right\}^k \tag{10}$$

Since this equation is the infinite geometrical progression, therefore,

$$I_{sample} = \frac{I_0 \cdot T^2 \cdot 10^{-A}}{1-(1-T)^2 \cdot 10^{-2A}} \tag{11}$$

The light intensity of blank (i.e. *A*=0) is then calculated as follows,

$$I_{blank} = \frac{I_0 \cdot T^2}{1-(1-T)^2} \tag{12}$$

Dividing I_{blank} / I_{sample} is given by

$$\frac{I_{blank}}{I_{sample}} = \frac{1-(1-T)^2 \cdot 10^{-2A}}{10^{-A} \cdot (1-(1-T)^2)} \tag{13}$$

This equation is general for CEAS and the absorption coefficient α is calculated as follows,

$$\alpha = \frac{T}{l} \cdot \frac{I_{blank} - I_{sample}}{I_{sample}} \tag{14}$$

If the reflection of the cavity mirror is close to 1 and the sample has low absorption and low scattering[1] . Eq. 14 can be recalculated as follows in order to show absorbance *A*,

$$\text{O.D.}(\lambda) = 0.434 \cdot T(\lambda) \cdot \frac{I_{blank}(\lambda) - I_{sample}(\lambda)}{I_{sample}(\lambda)} \tag{15}$$

(We use the following relation, $A = 0.434\alpha \cdot l$ (=O.D.), and we introduced the variable λ since samples and optics are depending on the wavelength).

Thus, the absorbance for homogenous solutions such as Venus protein shown in Fig. 2 was calculated by eq. 15. However, eq.15 is invalid if the sample has scattering such as cells. Therefore, for cellular measurements, we used the value I_{blank} / I_{sample}, which meant the inverse of transparency of the sample.

Reference

1. Ouyang B, Jones RL. Understanding the sensitivity of cavity-enhanced absorption spectroscopy: pathlength enhancement versus noise suppression. Appl Phys B. 2012;109: 581–591. doi:10.1007/s00340-012-5178-3
